# Development of a fourth-order compact finite difference scheme for simulation of simulated-moving-bed process

**DOI:** 10.1038/s41598-020-64562-8

**Published:** 2020-05-08

**Authors:** Chuanyi Yao, Yanjuan Zhang, Jinliang Chen, Xueping Ling, Keju Jing, Yinghua Lu, Enguo Fan

**Affiliations:** 1 Department of Chemical and Biochemical Engineering, College of Chemistry and Chemical Engineering, Xiamen University, Xiamen, 361005 China; 2 The Key Lab for Synthetic Biotechnology of Xiamen City, Xiamen University, Xiamen, 361005 China; 3 Department of Microbiology and Parasitology, Institute of Basic Medical Sciences, Chinese Academy of Medical Sciences/School of Basic Medicine, Peking Union Medical College, Beijing, China; 4 College of Chemistry and Chemical Engineering, Linyi University, Linyi, 276005 P.R. China

**Keywords:** Chemical engineering, Chemical engineering

## Abstract

A fourth-order compact finite difference scheme was developed to solve the model equation of simulated moving bed, which has a boundary condition that is updated along the calculation process and cannot be described as an explicit function of time. Two different methods, direct method and pseudo grid point method, were proposed to deal with the boundary condition. The high accuracy of the two methods was confirmed by a case study of solving an advection-diffusion equation with exact solution. The developed compact finite difference scheme was then used to simulate the SMB processes for glucose-fructose separation and enantioseparation of 1,1′-bi-2-naphtol. It was found that the simulated results fit well with the experimental data. Furthermore, the developed method was further combined with the continuous prediction method to shorten the computational time and the results showed that, the computational time can be saved about 45%.

## Introduction

The simulated moving bed (SMB) is a continuous preparative chromatography technique that has been widely used in various industries including petroleum^1,2^, food^3,4^, pharmaceutical^5–10^ and biotechnology^11–13^ to separate structurally similar compounds. To obtain an optimal operation conditions, a mathematical model is usually desired as it plays an essential role in the design and optimization of SMB process. Among them, the transport-dispersive model with linear driving force (LDF) approximation for describing mass transfer resistance between the mobile phase and the solid phase is believed to be the most widely used one with the following model equation^14,15^:

1 $$\frac{\partial c}{\partial t}+v\frac{\partial c}{\partial x}={D}_{{\rm{a}}}+\frac{{\partial }^{2}c}{\partial {x}^{2}}-\frac{1-{\varepsilon }_{{\rm{b}}}}{{\varepsilon }_{{\rm{b}}}}{k}_{{\rm{e}}}({q}^{\ast }-q)$$

2 $$\frac{\partial q}{\partial t}={k}_{{\rm{e}}}({q}^{\ast }-q)$$

This model equation is solved for every switching period to obtain the concentration profiles in the liquid and solid phase at any time. The initial condition of each switching period depends on the concentration profile in the columns at the end of the former switching period, and for the first switching period, the initial condition is:

3 $$t=0,c=q=0$$

The boundary conditions at the column inlet (*x* = 0) and outlet (*x* = *L*) are:

4 $$x=0,\frac{\partial c}{\partial x}=\frac{v}{{D}_{{\rm{a}}}}(c-{c}^{{\rm{in}}})$$

5 $$x=L,\frac{\partial c}{\partial x}=0$$

The mathematical SMB model equations have been solved using various methods such as the finite element^16^, finite difference^17^, finite volume (especially WENO)^14,18–22^, wavelet collocation^15^, as well as the space time conservation element and solution element (CE/SE) method^14,23^. However, no matter the method used, the accuracy and efficiency are usually in a contradict manner, i.e. a method with higher accuracy generally requires extensive calculations and *vice verse*. Therefore, developing a new method with high accuracy and efficiency, or at least making a suitable compromise between them is still a major issue for the design and optimization of the SMB processes.

The compact finite difference scheme (CFDS) attracts a great attention in the last twenty years^24–26^. In conventional finite difference scheme, the central difference with 2nd order precision needs 3 grid points. To construct a 4th order approximation, at least 5 grid points are necessary^27^, which would inevitably increase computation time greatly and add complexity for handling the boundary condition. However, CFDS can give a 4th order scheme by using only 3 grid points, which is achieved by coupling with the original governing equation^28^. That means CFDS combines a higher precision and fewer computation. However, the situation that derivation of CFDS needs governing equation makes it difficult to generalize. Usually the CFDS is constructed in a case by case manner, which is prohibitive for containing tedious mathematical treatment. Nevertheless, CFDS has been successfully used to solve numerous equations including convection-diffusion equation^29–35^, heat equation^25,36,37^, Gross-Pitaevskii equation^38^ and Helmholtz equation^39^. However, to the best of our knowledge, there is no report yet to solve the model equation of SMB using CFDS.

The SMB model equations are typical parabolic equation with Neumann boundary conditions. For this kind of equations, Mohebbi & Dehghan and Cao *et al*. have proposed to use the 4th-order CFDS^29,32^. But it should be addressed that in their methods, the boundary condition is an explicit function of time, and the derivative of this function is required to deal with the boundary condition. While for the SMB equations, as shown in Eqs. 1–5, the value of *c*^in^ in Eq. 4 is updated continuously with the calculation and as a consequence, the function of *c*^in^ verse *t* cannot be explicitly described, which makes it impossible to obtain the derivative of *c*^in^. So the methods reported in literature^29,32^ cannot be used to solve the SMB model equations.

To develop a 4th-order CFDS that can be used to solve the model equation of SMB, in the present work we compared two different methods for the handling of the boundary conditions. The accuracy of the developed method was examined by solving an equation with exact solution, and the computational efficiency was compared with the central difference scheme as well as the space-time solution element/conservation element method.

## The Fourth-Order Compact Finite Difference Scheme

By discretization on spatial domain, the Eqs. 1–2 can be transformed to a set of ordinary differential equations with time as an independent variable that can be solved by traditional methods, such as the “odeint” method in Scipy modula of Python. We thus first present the compact finite difference scheme for the spatial derivatives by considering the partial differential equation as follows:

6 $$-{D}_{{\rm{a}}}\frac{{\partial }^{2}c(x,t)}{\partial {x}^{2}}+v\frac{\partial c(x,t)}{\partial x}=f(x,t)$$

with boundary conditions:

7 $$\frac{\partial c(0,t)}{\partial x}={g}_{1}(t)$$

8 $$\frac{\partial c(L,t)}{\partial x}={g}_{2}(t)$$

The spatial domain [0, *L*] was discretized as follows:

9 $${x}_{i}=(i-1)h,i=1,2,\,\cdots ,\,n$$

with a constant step size *h* = *L*/(*n* − 1).

For the interior points, Mohebbi and Dehghan^32^ have derived the 4th order CFDS as below (the derivation can also be found in the Supplemental Materials):

10 $$\left[-\left({D}_{a}+\frac{{h}^{2}{v}^{2}}{12{D}_{a}}\right){\delta }_{x}^{2}+v{\delta }_{x}^{1}\right]c({x}_{i},t)=\left(\frac{{h}^{2}}{12}{\delta }_{x}^{2}-\frac{{h}^{2}v}{12{D}_{a}}{\delta }_{x}^{1}+1\right)f({x}_{i},t)$$

To construct the 4th order equations for the boundary points *x*_1_ and *x*_*n*_, the forward and backward difference schemes were used by denoting ${\Delta }_{jx}^{i}c(x,t)$ and ${\nabla }_{jx}^{i}c(x,t)$ as the forward and backward difference schemes of order *j* for the *i*th derivatives of *c*(*x*, *t*) about *x*, respectively. The formulas for *i* = 1, 2 and *j* = 1, 2 can be easily obtained from the Taylor expansions of *c*(*x*, *t*) at the points adjacent to *x*_*i*_ and are listed in Table 1, some of them can also be obtained from literature^27^.

**Table 1:** The forward and backward difference schemes of order 1 and 2 for the 1st and 2nd derivatives of *c* about *x*.

Symbol	Derivative	Truncation error	Formula
${\Delta }_{1x}^{1}c({x}_{i},t)$	$\frac{\partial c({x}_{i},t)}{\partial x}$	*O*(*h*)	$\frac{-c({x}_{i},\,t)+c({x}_{i+1},\,t)}{h}$
${\Delta }_{2x}^{1}c({x}_{i},t)$	$\frac{\partial c({x}_{i},t)}{\partial x}$	*O*(*h*^2^)	$\frac{-3c({x}_{i},\,t)+4c({x}_{i+1},\,t)-c({x}_{i+2},\,t)}{2h}$
${\Delta }_{1x}^{2}c({x}_{i},t)$	$\frac{{\partial }^{2}c({x}_{i},t)}{\partial {x}^{2}}$	*O*(*h*)	$\frac{c({x}_{i},\,t)-2c({x}_{i+1},\,t)+c({x}_{i+2},\,t)}{{h}^{2}}$
${\Delta }_{2x}^{2}c({x}_{i},t)$	$\frac{{\partial }^{2}c({x}_{i},t)}{\partial {x}^{2}}$	*O*(*h*^2^)	$\frac{2c({x}_{i},\,t)-5c({x}_{i+1},\,t)+4c({x}_{i+2},\,t)-c({x}_{i+3},\,t)}{{h}^{2}}$
${\nabla }_{1x}^{1}c({x}_{i},t)$	$\frac{\partial c({x}_{i},t)}{\partial x}$	*O*(*h*)	$\frac{-c({x}_{i-1},\,t)+c({x}_{i},\,t)}{h}$
${\nabla }_{2x}^{1}c({x}_{i},t)$	$\frac{\partial c({x}_{i},t)}{\partial x}$	*O*(*h*^2^)	$\frac{c({x}_{i-2},\,t)-4c({x}_{i-1},\,t)+3c({x}_{i},\,t)}{2h}$
${\nabla }_{1x}^{2}c({x}_{i},t)$	$\frac{{\partial }^{2}c({x}_{i},t)}{\partial {x}^{2}}$	*O*(*h*)	$\frac{c({x}_{i-2},\,t)-2c({x}_{i-1},\,t)+c({x}_{i},\,t)}{{h}^{2}}$
${\nabla }_{2x}^{2}c({x}_{i},t)$	$\frac{{\partial }^{2}c({x}_{i},t)}{\partial {x}^{2}}$	*O*(*h*^2^)	$\frac{-c({x}_{i-3},\,t)+4c({x}_{i-2},\,t)-5c({x}_{i-1},\,t)+2c({x}_{i},\,t)}{{h}^{2}}$

Two methods for handling of the boundary points were proposed here. In the first method, the derivatives at the boundary points were approximated by the forward or backward finite difference schemes directly thus named as “direct method”. While in the second method, a pseudo grid point (*x*_0_) was assumed to be exist at the left side of the boundary point *x*_1_, and on the contrary, a pseudo point (*x*_*n*+1_) is located at the right side of *x*_*n*_. So the second method was named as “pseudo grid point method”.

### Direct method

The equation dealing with the left boundary was deduced first. With the Taylor expansions of *c*(*x*_2_, *t*) and *c*(*x*_3_, *t*), the first derivative of *c*(*x*, *t*) at *x*_1_ can be expressed as:

11 $$\frac{\partial c({x}_{1},t)}{\partial x}={\Delta }_{2x}^{1}c({x}_{1},t)+\frac{{h}^{2}}{3}\frac{{\partial }^{3}c({x}_{1},t)}{\partial {x}^{3}}+\frac{{h}^{3}}{4}\frac{{\partial }^{4}c({x}_{1},t)}{\partial {x}^{4}}+O({h}^{4})$$

To construct a 4th order formula for $\partial c({x}_{1},\,t)/\partial x$, an approximation of 2nd order for ${\partial }^{3}c({x}_{1},\,t)/\partial {x}^{3}$ is needed, but for ${\partial }^{4}c({x}_{1},\,t)/\partial {x}^{4}$, the 1st order approximation is enough. By use of the control equation, Eq. 6, we have:

12 $$\frac{{\partial }^{3}c({x}_{i},t)}{\partial {x}^{3}}=\frac{v}{{D}_{a}}\frac{{\partial }^{2}c({x}_{i},t)}{\partial {x}^{2}}-\frac{1}{{D}_{a}}\frac{\partial f({x}_{i},t)}{\partial x}$$

13 $$\frac{{\partial }^{4}c({x}_{i},t)}{\partial {x}^{4}}=\frac{{v}^{2}}{{D}_{a}^{2}}\frac{{\partial }^{2}c({x}_{i},t)}{\partial {x}^{2}}-\frac{v}{{D}_{a}^{2}}\frac{\partial f({x}_{i},t)}{\partial x}-\frac{1}{{D}_{a}}\frac{{\partial }^{2}f({x}_{i},t)}{\partial {x}^{2}}$$

Using Eqs. 12–13, and substituting the derivatives by the forward difference schemes (Table 1) with appropriate order, the following equations are obtained:

14 $$\frac{{\partial }^{3}c({x}_{1},t)}{\partial {x}^{3}}=\frac{v}{{D}_{a}}{\Delta }_{2x}^{2}c({x}_{1},t)-\frac{1}{{D}_{a}}{\Delta }_{2x}^{1}f({x}_{1},t)+O({h}^{2})$$

15 $$\frac{{\partial }^{4}c({x}_{1},t)}{\partial {x}^{4}}==\frac{{v}^{2}}{{D}_{a}^{2}}{\Delta }_{1x}^{2}c({x}_{1},t)-\frac{v}{{D}_{a}^{2}}{\Delta }_{1x}^{1}f({x}_{1},t)-\frac{1}{{D}_{a}}{\Delta }_{1x}^{2}f({x}_{1},t)+O(h)$$

Combining Eqs. 14–15, Eq. 11 and the boundary condition in Eq. 7 gives:

16 $$\left({\Delta }_{2x}^{1}+\frac{{h}^{2}v}{3{D}_{a}}{\Delta }_{2x}^{2}+\frac{{h}^{3}{v}^{2}}{4{D}_{a}^{2}}{\Delta }_{1x}^{2}\right)c({x}_{1},t)=\frac{{h}^{2}}{{D}_{a}}\left(\frac{1}{3}{\Delta }_{2x}^{1}+\frac{hv}{4{D}_{a}}{\Delta }_{1x}^{1}+\frac{h}{4}{\Delta }_{1x}^{2}\right)f({x}_{1},t)+{g}_{1}(t)$$

Similarly, for the right boundary conditions, it can be obtained:

17 $$\left({\nabla }_{2x}^{1}+\frac{{h}^{2}v}{3{D}_{a}}{\nabla }_{2x}^{2}-\frac{{h}^{3}{v}^{2}}{4{D}_{a}^{2}}{\nabla }_{1x}^{2}\right)c({x}_{n},t)=\frac{{h}^{2}}{{D}_{a}}\left(\frac{1}{3}{\nabla }_{2x}^{1}-\frac{hv}{4{D}_{a}}{\nabla }_{1x}^{1}-\frac{h}{4}{\nabla }_{1x}^{2}\right)f({x}_{n},t)+{g}_{2}(t)$$

### Pseudo grid point method

For the left boundary point, it is assumed that there exists a pseudo grid point *x*_0_ at the left hand of *x*_1_. Then *x*_1_ can be looked as an interior point, and by using Eq. (10) the following equation was obtained:

18 $$\left[-\left({D}_{a}+\frac{{h}^{2}{v}^{2}}{12{D}_{a}}\right){\delta }_{x}^{2}+v{\delta }_{x}^{1}\right]c({x}_{1},t)=\left(\frac{{h}^{2}}{12}{\Delta }_{2x}^{2}-\frac{{h}^{2}v}{12{D}_{a}}{\Delta }_{2x}^{1}+1\right)f({x}_{1},t)$$

It should be emphasized that, in the right hand of Eq. (18), only the forward difference schemes were used for approximation of the derivatives of *f*(*x*_1_, *t*). This is because the partial difference of *c* about time was included in *f*, $\partial c(x,\,t)/\partial t$ is meaningless at *x*_0_. Nevertheless, the replacement of central difference schemes (${\delta }_{x}^{1}$ and ${\delta }_{x}^{2}$) by forward difference schemes (${\Delta }_{2x}^{1}$ and ${\Delta }_{2x}^{2}$) will not lead to the loss of accuracy, because all these four difference schemes have an accuracy of 2nd order.

In Eq. (18), *c*(*x*_0_, *t*) is implicitly included in the operators ${\delta }_{x}^{1}$ and ${\delta }_{x}^{2}$. It needs be eliminated by using the boundary condition Eq.(7). For this purpose, the first order and third order of *c*(*x*_1_, *t*) are expressed as follows:

19 $$\frac{\partial c({x}_{1},t)}{\partial x}={\delta }_{x}^{1}c({x}_{1},t)-\frac{{h}^{2}}{6}\frac{{\partial }^{3}c({x}_{1},t)}{\partial {x}^{3}}+O({h}^{4})$$

20 $$\frac{{\partial }^{3}c({x}_{1},t)}{\partial {x}^{3}}=\frac{v}{{D}_{a}}{\delta }_{x}^{2}c({x}_{1},t)-\frac{1}{{D}_{a}}{\Delta }_{2x}^{1}f({x}_{1},t)+O({h}^{2})$$

where Eq.(19) can be obtained by using the Taylor expansions at *c*(*x*_1_, *t*) and *c*(*x*_2_, *t*), while Eq. (20) is similar to Eq. (14) except that the operator ${\delta }_{x}^{2}$ is used to substitute ${\Delta }_{2x}^{2}$, both of them have a truncation error of *O*(*h*^2^).

Combining Eqs. 19–20, Eq. 11 and omitting the error term gives:

21 $${\delta }_{x}^{1}c({x}_{1},t)-\frac{{h}^{2}}{6}\left[\frac{v}{{D}_{a}}{\delta }_{x}^{2}c({x}_{1},t)-\frac{1}{{D}_{a}}{\Delta }_{2x}^{1}f({x}_{1},t)\right]={g}_{1}(t)$$

By combining Eq. 18 and Eq. 21 to eliminate $c({x}_{0},\,t)$, one obtains:

22 $$\begin{array}{rcl}\frac{{h}^{2}{v}^{2}-12{D}_{a}^{2}}{6{D}_{a}h+2{h}^{2}v}{\Delta }_{1x}^{1}c({x}_{1},t) & = & \left(\frac{{h}^{2}}{12}{\Delta }_{2x}^{2}+\frac{4{D}_{a}h+{h}^{2}v}{12{D}_{a}+4hv}{\Delta }_{2x}^{1}+1\right)f({x}_{1},t)\\  &  & -\frac{12{D}_{a}^{2}+{h}^{2}{v}^{2}+6{D}_{a}hv}{6{D}_{a}h+2{h}^{2}v}{g}_{1}(t)\end{array}$$

The right boundary conditions can be handled in a similar way, except that the backward difference schemes are used, the finally obtained equation is as follows:

23 $$\begin{array}{rcl}\frac{12{D}_{a}^{2}-{h}^{2}{v}^{2}}{6{D}_{a}h-2{h}^{2}v}{\nabla }_{1x}^{1}c({x}_{N},t) & = & \left(\frac{{h}^{2}}{12}{\nabla }_{2x}^{2}-\frac{4{D}_{a}h-{h}^{2}v}{12{D}_{a}-4hv}{\nabla }_{2x}^{1}+1\right)f({x}_{N},t)\\  &  & +\,\frac{12{D}_{a}^{2}+{h}^{2}{v}^{2}-6{D}_{a}hv}{6{D}_{a}h-2{h}^{2}v}{g}_{2}(t)\end{array}$$

### Solution of SMB model equations

For the model equation of SMB process, the function *f*(*x*, *t*) in Eq. (6) is as follows:

24 $$f(x,t)=-\frac{\partial c(x,\,t)}{\partial t}-{k}_{eff}\frac{(1-)}{\varepsilon }({q}^{\ast }-q)$$

And the boundary conditions are:

25 $${g}_{1}(t)=\frac{v}{{D}_{a}}(c-{c}^{{\rm{in}}})$$

26 $${g}_{2}(t)=0$$

While using the “direct method” for handling the boundary conditions, Eqs. 24–26 are introduced into Eq. 11 and Eqs. 16–17. Then the SMB model equations are transformed to a system of ordinary equations as follows:

27 $${\boldsymbol{A}}{{\boldsymbol{c}}}^{\text{'}}(t)={\boldsymbol{Bc}}(t)-{k}_{eff}\frac{(1-\varepsilon )}{\varepsilon }{\boldsymbol{A}}[{{\boldsymbol{q}}}^{\ast }(t)-{\boldsymbol{q}}(t)]+{\boldsymbol{g}}(t)$$

28 $${\boldsymbol{q}}{\boldsymbol{{\prime} }}(t)={k}_{eff}[{{\boldsymbol{q}}}^{\ast }(t)-{\boldsymbol{q}}(t)]$$

in which

29 $${\boldsymbol{c}}(t)={[{c}_{1}(t),{c}_{2}(t),\cdots ,{c}_{N}(t)]}^{{\rm{T}}}$$

30 $${\boldsymbol{q}}(t)={[{q}_{1}(t),{q}_{2}(t),\cdots ,{q}_{N}(t)]}^{{\rm{T}}}$$

31 $${{\boldsymbol{q}}}^{\ast }(t)={[{q}_{1}^{\ast }(t),{q}_{2}^{\ast }(t),\cdots ,{q}_{N}^{\ast }(t)]}^{{\rm{T}}}$$

32 $${\boldsymbol{g}}(t)={[-v{c}^{{\rm{in}}}/h,0,\cdots ,0]}^{{\rm{T}}}$$

33 $${\boldsymbol{A}}=\left[\begin{array}{ccccc}-\frac{1}{4}-\frac{hv}{4{D}_{a}} & \frac{1}{6}+\frac{hv}{4{D}_{a}} & \frac{1}{12} &  & \\ \frac{1}{12}+\frac{hv}{24{D}_{a}} & \frac{5}{6} & \frac{1}{12}-\frac{hv}{24{D}_{a}} &  & \\  & \ddots  & \ddots  & \ddots  & \\  &  & \frac{1}{12}+\frac{hv}{24{D}_{a}} & \frac{5}{6} & \frac{1}{12}-\frac{hv}{24{D}_{a}}\\  &  & -\,\frac{1}{12} & -\,\frac{1}{6}+\frac{hv}{4{D}_{a}} & \frac{1}{4}-\frac{hv}{4{D}_{a}}\end{array}\right]$$

34 $${\boldsymbol{B}}=\left[\begin{array}{ccccc}\frac{3{D}_{a}}{2{h}^{2}}+\frac{v}{3h}-\frac{{v}^{2}}{4{D}_{a}} & -\frac{2{D}_{a}}{{h}^{2}}+\frac{5v}{3h}+\frac{{v}^{2}}{2{D}_{a}} & \frac{{D}_{a}}{2{h}^{2}}-\frac{4v}{3h}-\frac{{v}^{2}}{4{D}_{a}} & \frac{v}{3h} & \\ \frac{{D}_{a}}{{h}^{2}}+\frac{v}{2h}+\frac{{v}^{2}}{12{D}_{a}} & -\frac{2{D}_{a}}{{h}^{2}}-\frac{{v}^{2}}{6{D}_{a}} & \frac{{D}_{a}}{{h}^{2}}-\frac{v}{2h}+\frac{{v}^{2}}{12{D}_{a}} &  & \\  & \ddots  & \ddots  & \ddots  & \\  &  & \frac{{D}_{a}}{{h}^{2}}+\frac{v}{2h}+\frac{{v}^{2}}{12{D}_{a}} & -\frac{2{D}_{a}}{{h}^{2}}-\frac{{v}^{2}}{6{D}_{a}} & \frac{{D}_{a}}{{h}^{2}}-\frac{v}{2h}+\frac{{v}^{2}}{12{D}_{a}}\\  & \frac{v}{3h} & -\frac{{D}_{a}}{2{h}^{2}}-\frac{4v}{3h}+\frac{{v}^{2}}{4{D}_{a}} & \frac{2{D}_{a}}{{h}^{2}}+\frac{5v}{3h}-\frac{{v}^{2}}{2{D}_{a}} & -\frac{3{D}_{a}}{2{h}^{2}}-\frac{2v}{3h}+\frac{{v}^{2}}{4{D}_{a}}\end{array}\right]$$

If the pseudo grid point method was used to treat the boundary conditions, the resulted equations have the same pattern as Eqs. 27–28, but the vectors ***g***(*t*), ***A*** and ***B*** are different from Eqs. 32–34, which are as follows:

35 $${\boldsymbol{g}}(t)={\left[\left(\frac{6v}{h}+\frac{h{v}^{3}}{2{D}_{a}^{2}}+\frac{3{v}^{2}}{{D}_{a}}\right){c}^{{\rm{in}}},0,\cdots ,0\right]}^{{\rm{T}}}$$

36 $${\boldsymbol{A}}=\left[\begin{array}{ccccc}2+\frac{19\,hv}{24{D}_{a}} & \frac{3}{4}+\frac{hv}{12{D}_{a}} & \frac{1}{2}+\frac{5\,hv}{24{D}_{a}} & -\frac{1}{4}-\frac{hv}{12{D}_{a}} & \\ \frac{1}{12}+\frac{hv}{24{D}_{a}} & \frac{5}{6} & \frac{1}{12}-\frac{hv}{24{D}_{a}} &  & \\  & \ddots  & \ddots  & \ddots  & \\  &  & \frac{1}{12}+\frac{hv}{24{D}_{a}} & \frac{5}{6} & \frac{1}{12}-\frac{hv}{24{D}_{a}}\\  & \frac{1}{4}-\frac{hv}{12{D}_{a}} & -\,\frac{1}{2}+\frac{5\,hv}{24{D}_{a}} & -\,\frac{3}{4}+\frac{hv}{12{D}_{a}} & -\,2+\frac{19\,hv}{24{D}_{a}}\end{array}\right]$$

37 $${\boldsymbol{B}}=\left[\begin{array}{ccccc}-\frac{6{D}_{a}}{{h}^{2}}-\frac{6v}{h}-\frac{5{v}^{2}}{2{D}_{a}}-\frac{h{v}^{3}}{2{D}_{a}^{2}} & \frac{6{D}_{a}}{{h}^{2}}-\frac{{v}^{2}}{2{D}_{a}} &  &  & \\ \frac{{D}_{a}}{{h}^{2}}+\frac{v}{2h}+\frac{{v}^{2}}{12{D}_{a}} & -\frac{2{D}_{a}}{{h}^{2}}-\frac{{v}^{2}}{6{D}_{a}} & \frac{{D}_{a}}{{h}^{2}}-\frac{v}{2h}+\frac{{v}^{2}}{12{D}_{a}} &  & \\  & \ddots  & \ddots  & \ddots  & \\  &  & \frac{{D}_{a}}{{h}^{2}}+\frac{v}{2h}+\frac{{v}^{2}}{12{D}_{a}} & -\frac{2{D}_{a}}{{h}^{2}}-\frac{{v}^{2}}{6{D}_{a}} & \frac{{D}_{a}}{{h}^{2}}-\frac{v}{2h}+\frac{{v}^{2}}{12{D}_{a}}\\  &  &  & -\frac{6{D}_{a}}{{h}^{2}}+\frac{{v}^{2}}{2{D}_{a}} & \frac{6{D}_{a}}{{h}^{2}}-\frac{{v}^{2}}{2{D}_{a}}\end{array}\right]$$

The resulted ordinary differential equations are solved by the method of “odeint” in Scipy modula of python 3.6.4. All the calculations in this work were conducted on a PC with CPU 3.60 GHz, RAM 16.0 GB.

## Results and Discussion

### Advection-diffusion equation with analytical solution

To test the accuracy of the CFDS developed in this work, the transient one-dimensional advection-diffusion equation was first solved as follows^29,32,40^:

38 $$\frac{\partial c(x,t)}{\partial t}=-v\frac{\partial c(x,t)}{\partial x}+{D}_{a}\frac{{\partial }^{2}c(x,t)}{\partial {x}^{2}}$$

with initial condition:

39 $$c(x,0)=\exp \left[-\frac{{(x-0.2)}^{2}}{{D}_{a}}\right]$$

and boundary conditions:

40 $$\frac{\partial c(0,t)}{\partial x}={{\rm{g}}}_{1}({\rm{t}})=\frac{2(0.2+vt)}{{D}_{a}(4t+1)\sqrt{4t+1}}\exp \left[-\frac{{(0.2+vt)}^{2}}{{D}_{a}(4t+1)}\right]$$

41 $$\frac{\partial c(1,t)}{\partial x}={{\rm{g}}}_{2}({\rm{t}})=-\frac{2(0.8-vt)}{{D}_{a}(4t+1)\sqrt{4t+1}}\exp \left[-\frac{{(0.8-vt)}^{2}}{{D}_{a}(4t+1)}\right]$$

This equation governs the distribution of concentration *c* at position *x* (1 ≥ *x* ≥ 0) at time *t* in a fluid moving with fixed advective speed *v* and subject to diffusion governed by the coefficient *D*_*a*_. The initial condition is a Gaussian pulse of unit height centered at *x* = 0.2. The exact solution of the equation is as follows:

42 $$c(x,t)=\frac{1}{\sqrt{4t+1}}\exp \left[-\frac{{(x-0.2-vt)}^{2}}{{D}_{a}(4t+1)}\right]$$

For this equation, the ordinary equation Eq. 27 is simplified to:

43 $${\boldsymbol{A}}{{\boldsymbol{c}}}^{\text{'}}(t)={\boldsymbol{Bc}}(t)+{\boldsymbol{g}}(t)$$

in which, while using the “direct method” to treat the boundary conditions,

44 $${\boldsymbol{g}}(t)={[{D}_{a}{g}_{1}(t)/h,0,\cdots ,0,{D}_{a}{g}_{2}(t)/h]}^{{\rm{T}}}$$

***A*** is the same as in Eq. 33, and ***B*** is as follows:

45 $${\boldsymbol{B}}=\left[\begin{array}{ccccc}\frac{3{D}_{a}}{2{h}^{2}}-\frac{2v}{3h}-\frac{{v}^{2}}{4{D}_{a}} & -\frac{2{D}_{a}}{{h}^{2}}+\frac{5v}{3h}+\frac{{v}^{2}}{2{D}_{a}} & \frac{{D}_{a}}{2{h}^{2}}-\frac{4v}{3h}-\frac{{v}^{2}}{4{D}_{a}} & \frac{v}{3h} & \\ \frac{{D}_{a}}{{h}^{2}}+\frac{v}{2h}+\frac{{v}^{2}}{12{D}_{a}} & -\frac{2{D}_{a}}{{h}^{2}}-\frac{{v}^{2}}{6{D}_{a}} & \frac{{D}_{a}}{{h}^{2}}-\frac{v}{2h}+\frac{{v}^{2}}{12{D}_{a}} &  & \\  & \ddots  & \ddots  & \ddots  & \\  &  & \frac{{D}_{a}}{{h}^{2}}+\frac{v}{2h}+\frac{{v}^{2}}{12{D}_{a}} & -\frac{2{D}_{a}}{{h}^{2}}-\frac{{v}^{2}}{6{D}_{a}} & \frac{{D}_{a}}{{h}^{2}}-\frac{v}{2h}+\frac{{v}^{2}}{12{D}_{a}}\\  & \frac{v}{3h} & -\frac{{D}_{a}}{2{h}^{2}}-\frac{4v}{3h}+\frac{{v}^{2}}{4{D}_{a}} & \frac{2{D}_{a}}{{h}^{2}}+\frac{5v}{3h}-\frac{{v}^{2}}{2{D}_{a}} & -\frac{3{D}_{a}}{2{h}^{2}}-\frac{2v}{3h}+\frac{{v}^{2}}{4{D}_{a}}\end{array}\right]$$

when using the pseudo grid point method for treating the boundary conditions,

46 $${\boldsymbol{g}}(t)={\left[-\left(\frac{6{D}_{a}}{h}+\frac{h{v}^{2}}{2{D}_{a}}+3v\right){g}_{1}({\rm{t}}),0,\cdots ,0,-\left(\frac{6{D}_{a}}{h}+\frac{h{v}^{2}}{2{D}_{a}}+3v\right){g}_{2}({\rm{t}})\right]}^{{\rm{T}}}$$

***A*** is the same as in Eq. 36, and ***B*** is as follows:

47 $${\boldsymbol{B}}=\left[\begin{array}{ccccc}-\frac{6{D}_{a}}{{h}^{2}}+\frac{{v}^{2}}{2{D}_{a}} & \frac{6{D}_{a}}{{h}^{2}}-\frac{{v}^{2}}{2{D}_{a}} &  &  & \\ \frac{{D}_{a}}{{h}^{2}}+\frac{v}{2h}+\frac{{v}^{2}}{12{D}_{a}} & -\frac{2{D}_{a}}{{h}^{2}}-\frac{{v}^{2}}{6{D}_{a}} & \frac{{D}_{a}}{{h}^{2}}-\frac{v}{2h}+\frac{{v}^{2}}{12{D}_{a}} &  & \\  & \ddots  & \ddots  & \ddots  & \\  &  & \frac{{D}_{a}}{{h}^{2}}+\frac{v}{2h}+\frac{{v}^{2}}{12{D}_{a}} & -\frac{2{D}_{a}}{{h}^{2}}-\frac{{v}^{2}}{6{D}_{a}} & \frac{{D}_{a}}{{h}^{2}}-\frac{v}{2h}+\frac{{v}^{2}}{12{D}_{a}}\\  &  &  & -\frac{6{D}_{a}}{{h}^{2}}+\frac{{v}^{2}}{2{D}_{a}} & \frac{6{D}_{a}}{{h}^{2}}-\frac{{v}^{2}}{2{D}_{a}}\end{array}\right]$$

**Figure 1:**
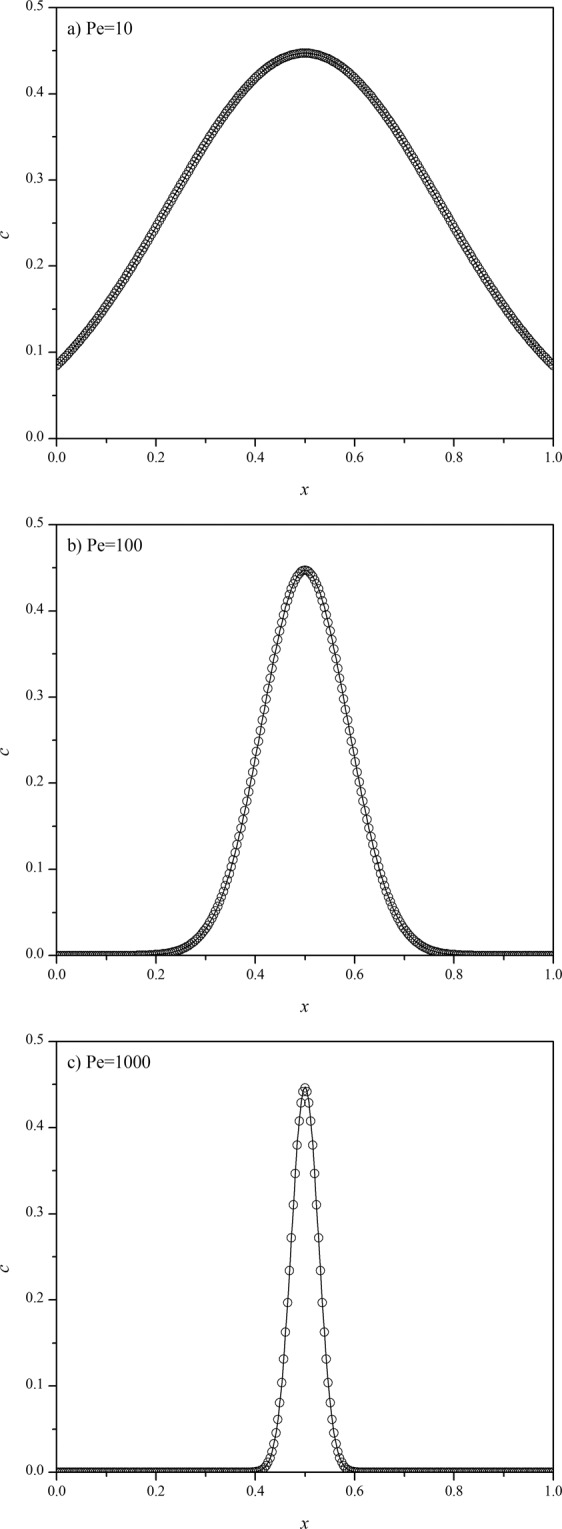
The exact solution (line) of the advection diffusion equation defined in Eqs. 38–41 and the solution (points) obtained by the compact finite difference method with direct method for handling the boundary conditions at *t* = 1, *v* = 0.3, *h* = 1/2^8^, and *Pe* = 10 (**a**), *Pe* = 100 (**b**), and *Pe* = 1000 (**c**).

**Figure 2:**
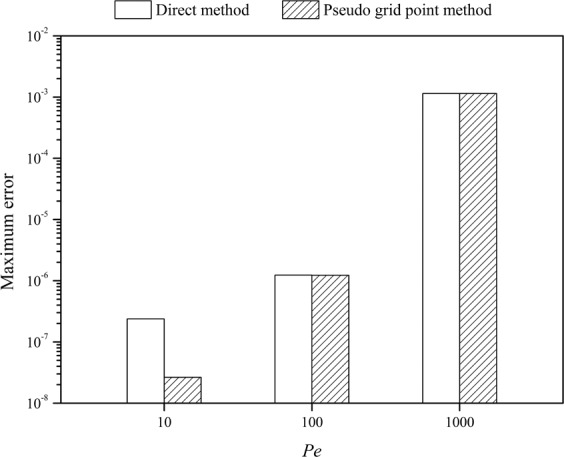
Comparison of the maximum error of the solution while using the direct method and pseudo grid point method for handling the boundary conditions. The advection diffusion equation defined in Eqs. 38–41 is solved at *t* = 1, *v* = 0.3, *h* = 1/2^8^ and different *Pe* numbers.

The advection-diffusion equation was solved by the CFDS with two different methods for handling the boundary conditions. It was found that both methods have a high accuracy. As an example, the solutions obtained by the direct method at final time *t* = 1 with *v* = 0.3, *h* = 1/2^8^, and different *Pe* numbers (*Pe* = *vL*/*D*_*a*_) are shown in Fig. 1. It can be observed that the calculated concentration profiles are in a good accordance with the exact solutions, even for a higher Peclet number of 1000 (Fig. 1c). When using the pseudo grid point method to handle the boundary conditions, the solution has no significant difference compared with that shown in Fig. 1. Then the maximum error was evaluated with the maximum difference between the concentrations obtained from CFDS and analytical solution while *x* ranging from 0 to 1, it was found appear at *x* = 0.5. The maximum errors of the two methods were shown in Fig. 2. Of course, for a fixed *h* value, the maximum error will increase with the increase of *Pe* number. This is reasonable because a higher *Pe* number results in a sharper peak, and as a consequence, a denser mesh (smaller *h* value) along with the *x*-axis is usually needed. Meanwhile, the two methods for handling the boundary condition have a similar accuracy at higher *Pe* numbers ( > 100), but at lower *Pe* number (say *Pe* = 10) the pseudo grid point method gives a higher accuracy than the direct method. So, the pseudo grid point method is recommended and hereinafter was used for further analysis. However, it should be noted that the CFDS presented in this work has a very high accuracy at lower *Pe* numbers, such as the maximum errors at *Pe* = 10 shown in Fig. 2 were 2.4×10^−7^ and 2.6×10^−8^ for the direct and pseudo grid point methods, respectively. So even the direct method is accurate enough for most of the applications.

**Figure 3:**
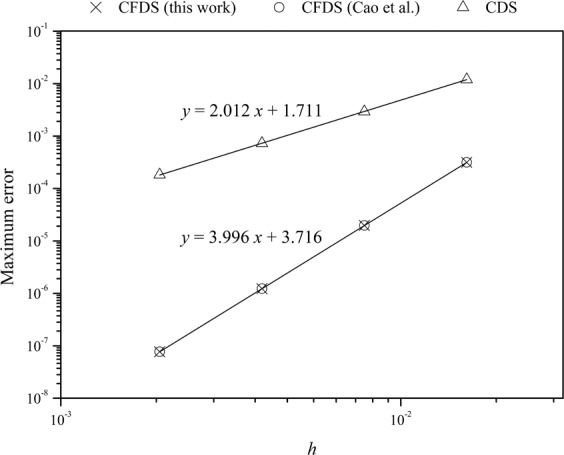
Double logarithmic plots of maximum error vs. *h* for central differential scheme (CDS) and the compact finite differential schemes (CFDS) presented in this work and Cao’s work^29^. Lines are obtained by linear regression. The advection diffusion equation defined in Eqs. 38–41 is solved at *t* = 1, *v* = 0.3, *Pe* = 100 and different *h* values.

To examine the convergence rate of the method developed above, the advection diffusion equation was solved at *h* = 1/2^6^, 1/2^7^, 1/2^8^, and 1/2^9^. For a method with *n*-order accuracy, the solution error (*E*) at any point is proportional to *h*^*n*^ and so the date log(*E*) vs. log(*h*) should be asymptotic to a straight line with slope *n*. Therefore, the solution errors at different *h* are shown in a double logarithmic plot (Fig. 3) by measuring the error at *x* = 0.5, which is the maximum error along the *x*-axis. It can be seen that the points have a good linear relationship. By linear regression, the slope was found to be 3.996, which thus confirms that the method developed in this work has a convergence rate of 4th-order. As a comparison, the central differential scheme^41^ was also used to solve this advection diffusion equation at different *h* values, and as expected, a convergence rate of 2-order was confirmed with a slope of 2.012 as shown in Fig. 3.

We also used the CFDS that was developed by Cao *et al*.^29^ to solve the equations defined in Eqs. 38–41. The results showed that the maximum errors at different *h* values are the same as that of our method (Fig. 3) thus suggesting that the two methods have a similar accuracy. Because in Cao′s method, the vector ***A*** in Eq. 33 is tri-diagonal (for detailed information, please refer to the original paper), this makes their methods favorable for the stability analysis. However, the differential function of boundary conditions, i.e. ***g***′(*t*), is needed, thus makes Cao’s method not possible for some applications, such as the SMB equation, where *c*^in^ is included in the boundary conditions (Eq. 4). Because *c*^in^ can only be obtained with calculation, its differential function and so ***g***′(*t*) cannot be obtained. This is our aim in the present work to develop a compact finite difference scheme to solve SMB equation. Of course we believe our method can also be used for other applications because the differential function of boundary conditions is not needed. However, we have to point out that the matrix ***A*** in Eq. 33 is not tri-diagonal thus the stability analysis becomes difficult. Nonetheless, the stability and small stencil of CFDS have been widely accepted. For the detailed discussion of the stability while using CFDS for solving the convection-diffusion equations, please refer to the literature.^29,32,42,43^

### Numerical solution of simulated-moving-bed model equation

We then sought to use the compact finite difference scheme to solve the model equation of simulated moving bed. Two SMB processes, glucose-fructose separation and enantioseparation of 1,1’-bi-2-naphtol, were used as the case studies. The parameters of the two systems were obtained from literature^4,7,44,45^ as summarized in Table 2. The adsorption isotherms of sugar separation process is nearly linear:

48 $${q}_{{\rm{A}}}^{\ast }=0.675{c}_{{\rm{A}}}$$

49 $${q}_{{\rm{B}}}^{\ast }=0.32{c}_{{\rm{B}}}+0.000457{c}_{{\rm{A}}}{c}_{{\rm{B}}}$$

while the adsorption isotherms of enantioseparation process is highly nonlinear:

50 $${q}_{{\rm{A}}}^{\ast }=\frac{3.73{c}_{{\rm{A}}}}{1+0.0466{c}_{{\rm{A}}}+0.0336{c}_{{\rm{B}}}}+\frac{0.3{c}_{{\rm{A}}}}{1+3{c}_{{\rm{A}}}+{c}_{{\rm{B}}}}$$

51 $${q}_{{\rm{B}}}^{\ast }=\frac{2.69{c}_{{\rm{B}}}}{1+0.0466{c}_{{\rm{A}}}+0.0336{c}_{{\rm{B}}}}+\frac{0.1{c}_{{\rm{B}}}}{1+3{c}_{{\rm{A}}}+{c}_{{\rm{B}}}}$$

**Table 2:** Parameters of the simulated moving bed processes for sugar (fructose-glucose) separation and enantioseparation of 1,1′-bi-2-naphtol.

Items	Sugar separation	Enantioseparation
Column	i.d. 2.6 cm × 52.07 cm	i.d. 2.6 cm × 10.5 cm
Configuration	2/2/2/2	2/2/2/2
Bed porosity	0.41	0.4
Switching time, min	16.39	2.75
Feed concentration, g L^−1^	${c}_{{\rm{A}}}=363,{c}_{{\rm{B}}}=322$	${c}_{{\rm{A}}}={c}_{{\rm{B}}}=2.9$
Mass transfer coefficient, min^−1^	${k}_{{\rm{e}},{\rm{A}}}=0.72,{k}_{{\rm{e}},{\rm{B}}}=0.9$	${k}_{{\rm{e}},{\rm{A}}}={k}_{{\rm{e}},{\rm{B}}}=6.0$
Flow rate in four zones, mL min^−1^	15.89, 11.0, 12.67, 9.1	56.83, 40.83, 44.47, 35.38
Apparent dispersion coefficient in four zones, cm^2^ min^−1^	1.105, 0.765, 0.881, 0.633	0.281, 0.202, 0.220, 0.175

In the simulation, the step sizes are set to be *h* = *L*/64 and *L*/40 for the sugar separation and enantioseparation processes respectively^14,15,23^. Furthermore, the criterion for achieving the cyclic steady state (CSS) is set to be that the maximum difference between the concentrations in two consecutive iterations is lower than 10^−4^ of feed concentration. It is found that the sugar separation process needs 91 switches for achieving CSS upon this criterion, while it is generally acknowledged that 80 switches are sufficient to reach CSS in this SMB process^4,15^. So it is believed that this criterion is appropriate and is used in the simulation of the two case studies.

**Figure 4:**
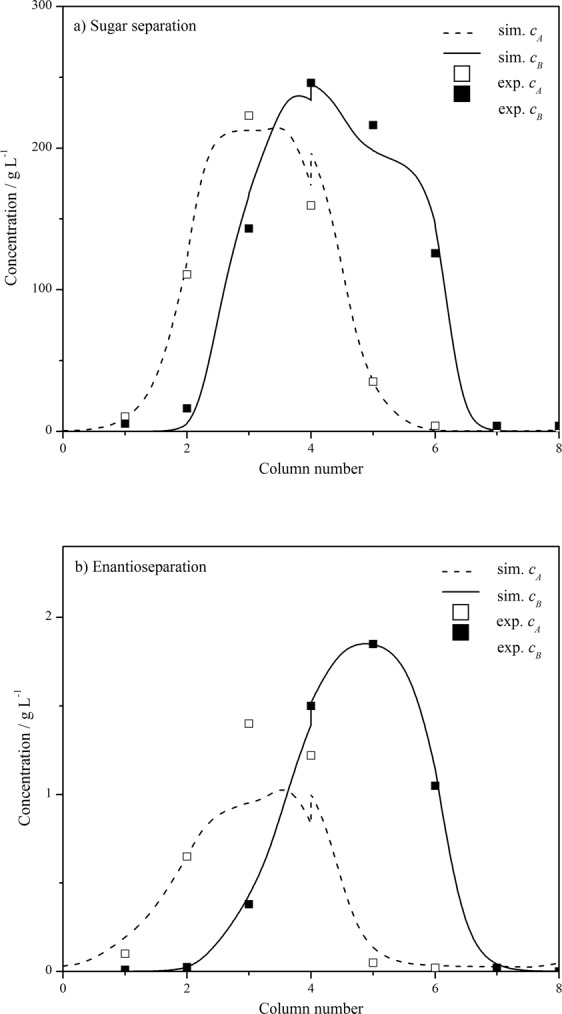
Concentration profiles of glucose-fructose separation (**a**) and enantioseparation (**b**) processes at middle of a switching period after reaching cyclic steady state calculated by compact finite difference method developed in this work. The experimental results are obtained from literature^4,44^.

The simulated concentration profiles at half of a switching period after reaching CSS are shown in Fig. 4. It can be seen that the simulation results fit the experimental data well for both the two SMB systems. For comparison, the space-time conservation element and solution element (CE/SE) method^23^ was also used to simulate the two SMB processes. The results are listed in Table 3. Obviously, the products purities and recoveries obtained by different methods are similar. But the calculation time of CFDS is much longer than that of CE/SE method. This is reasonable because a higher accuracy is usually accompanied by an expensive computation. So, for the applications where the simulation efficiency is desired, the CE/SE method is preferred, while for the applications where high accuracy is desired, the CFDS method is a very good option.

**Table 3:** Simulation results and calculation times of CFDS and CE/SE methods for two SMB processes of sugar (glucose-fructose) separation and enantioseparation of 1,1′-bi-2-naphtol.

SMB process	Simulation method	Purity, %		Recovery, %		Calculation time, s
		Ext.	Raf.	Ext.	Raf.	
Sugar separation	Experimental^4^	81.6	92.9	96.4	80.4	—
	CE/SE	90.5	97.3	97.8	88.3	58.4 ± 2
	CFDS	87.9	98.3	98.7	84.5	354 ± 5
Enantioseparation	Experimental^36^	93.0	96.2	99.1	94.1	—
	CE/SE	95.4	97.1	96.9	95.7	80.8 ± 3
	CFDS	93.6	96.8	96.8	93.4	1240 ± 30

In our previous work, a continuous prediction method was developed to improve the simulation efficiency of SMB process^46^. The key point of this continuous prediction method is the construction or prediction of the concentration at CSS using the concentrations in the last two iterations. The formula is as follows:

52 $${{\boldsymbol{u}}}_{{\rm{CSS}}}^{(m)}={{\boldsymbol{u}}}^{(m)}+\theta [{{\boldsymbol{u}}}^{(m)}-{{\boldsymbol{u}}}_{{\rm{CSS}}}^{(m-1)}]$$

**Figure 5:**
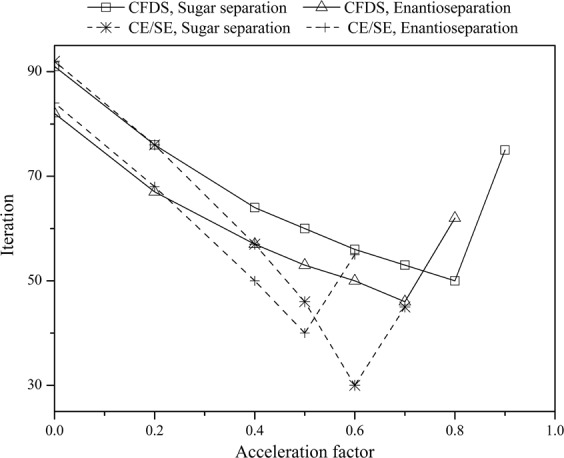
Dependence of iteration number needed for reaching cyclic steady state on acceleration factor while using the compact finite difference scheme (CFDS) or space-time conservation element and solution element method (CE/SE) to solve the model equation of SMB processes for separation of fructose and glucose and enatioseparation of 1,1′-bi-2-naphtol.

Here, state variable ***u*** contains the concentrations of two solutes in the mobile phase and in the stationary phase, i.e. *c*_A_, *c*_B_, *q*_A_, and *q*_B_. The superscript *m* means the *m*th iteration and the acceleration factor *θ* is an empirical parameter. Then, the predicted state variable, ${{\boldsymbol{u}}}_{{\rm{CSS}}}^{(m)}$, is used as the initial value for the next iteration. Through this method, the iterations needed to achieve CSS can be reduced and thus the calculation time can be shortened. So, the continuous prediction method was used to improve the efficiency of CFDS in this work. The iteration numbers needed to reach CSS at different *θ* values are shown in Fig. 5. It can be seen that the computational efficiencies of the two SMB processes were both significantly improved with the aid of continuous prediction method. For the sugar separation process, an acceleration factor *θ* = 0.8 will lead to an iteration number of 50 for reaching CSS. While without the continuous prediction method used, i.e. *θ* = 0, the iteration number is 91. Accordingly, the calculation time was saved about 45% due to the CPU time is proportional to the iteration number^46^. As for the enantioseparation process, the calculation time can also be saved about 44% with *θ* = 0.7 (46 iterations) compared to *θ* = 0 (82 iterations). But to our surprise, the optimum *θ* values are much higher than the value (*θ* = 0.5) that we have previously recommended^23,46^. For comparison, the dependence of iteration number on *θ* when using CE/SE method was also shown in Fig. 5. The optimum *θ* values are 0.5 and 0.6 for the enantioseparation and sugar separation processes respectively, which is in consistence with our previous results^46^. The optimum *θ* values in CFDS are shifted 0.2 higher than that in CE/SE method for both the two SMB processes (0.5 to 0.7 for enantioseparation and 0.6 to 0.8 for sugar separation processes). The reason for this shift is unclear, but it is reasonable to draw a conclusion that the optimum *θ* value depends on the methods that are used to solve the model equation, even for the same SMB process.

## Conclusion

In the present work, a fourth-order compact finite difference scheme was successfully developed to solve the advection diffusion equations with Neumann boundary conditions, which do not need the boundary conditions to be differentiable function. Two different methods, direct method and pseudo grid point method, were proposed and used to handle the boundary conditions. The higher accuracy of the compact finite difference scheme was confirmed by a case study with analytical solution. It was found that the pseudo grid point method results in a higher accuracy than the direct method when the *Pe* number is low (such as 10). But for a moderate and high *Pe* numbers (such as 100 and 1000), the two methods give the same accuracy.

It should be pointed out that although the CFDS method can be used to solve the SMB model equations, the calculation time is much longer than the space-time conservation element and solution element method. This problem, however, can be solved by use of the continuous prediction method, which improves the calculation efficiency of CFDS significantly meanwhile saves the calculation time about 45%. In summary, it is tempting to speculate that the combination of 4th-order compact finite difference scheme described in the present work and the continuous prediction method can be used for most of the SMB processes and has a wide application potential, especially when a higher accuracy is desired.

## Supplementary information

Supplementary information
