# Supplementary material for: Development of a fourth-order compact finite difference scheme for simulation of simulated-moving-bed process
Source: Sci Rep. 2020 May 8;10:7820. doi: 10.1038/s41598-020-64562-8 (PMC7210932; doi:10.1038/s41598-020-64562-8)
Supplement: Supplementary file 1 — Supplementary information [file 41598_2020_64562_MOESM1_ESM.docx]

Development of a fourth-order compact finite difference scheme for simulation of simulated-moving-bed process

Chuanyi Yao^a,b,*^, Yanjuan Zhang^c^, Jinliang Chen^a^, Xueping Ling^a,b^, Keju Jing^a,b^, Yinghua Lu^a,b^, Enguo Fan^c,d*^

^a^ *Department of Chemical and Biochemical Engineering, College of Chemistry and Chemical Engineering, Xiamen University, Xiamen, 361005, China*

^b^ *The Key Lab for Synthetic Biotechnology of Xiamen City, Xiamen University, Xiamen, 361005, China*

^c^ Department of Microbiology and Parasitology, Institute of Basic Medical Sciences, Chinese Academy of Medical Sciences/School of Basic Medicine, Peking Union Medical College, Beijing, China

^d^ College of Chemistry and Chemical Engineering, Linyi University, Linyi 276005, P. R. China

Correspondence

Chuanyi Yao, Department of Chemical and Biochemical Engineering, College of Chemistry and Chemical Engineering, Xiamen University, 361005 Xiamen, Fujian, China.

Email: cyao@xmu.edu.cn

Enguo Fan, Department of Microbiology and Parasitology, Institute of Basic Medical Sciences, Chinese Academy of Medical Sciences/School of Basic Medicine, Peking Union Medical College, Beijing, China

Email: enguo.fan@ibms.pumc.edu.cn

**Derivation of the 4th order CFDS for interior points (Eq.10 in the manuscript):**

For the differential equation as follow (Eq. 6 in the manuscript):

$-D_{a}\frac{\partial^{2}c(x,t)}{{\partial x}^{2}}+v\frac{\partial c(x,t)}{\partial x}=f(x,t)$ (1)

with boundary conditions:

$\frac{\partial c(0,t)}{\partial x}=g_{1}(t)$ (2)

$\frac{\partial c(L,t)}{\partial x}=g_{2}(t)$ (3)

The spatial domain $[0,L]$ was discretized as follows:

$x_{i}=\left( i-1 \right)h, i=1,2,\cdots,n$ (4)

with a constant step size $h=L/(n-1)$.

With the Taylor expansions of $c(x,t)$ at points $x_{i+1}$ and $x_{i-1}$:

$c\left( x_{i\pm1}, t \right)=c\left( x_{i}, t \right)\pm h\frac{\partial c\left( x_{i}, t \right)}{\partial x}+\frac{h^{2}}{2!}\frac{\partial^{2}c\left( x_{i}, t \right)}{{\partial x}^{2}}\pm\frac{h^{3}}{3!}\frac{\partial^{3}c\left( x_{i}, t \right)}{{\partial x}^{3}}+\frac{h^{4}}{4!}\frac{\partial^{4}c\left( x_{i}, t \right)}{{\partial x}^{4}}\pm\frac{h^{5}}{5!}\frac{\partial^{5}c\left( x_{i}, t \right)}{{\partial x}^{5}}+O\left( h^{6} \right)$ (5)

the first and second derivatives of $c(x,t)$ about $x$ can be easily obtained as:

$\frac{\partial c\left( x_{i}, t \right)}{\partial x}=\delta_{x}^{1}c\left( x_{i}, t \right)-\frac{h^{2}}{6}\frac{\partial^{3}c\left( x_{i}, t \right)}{{\partial x}^{3}}+O\left( h^{4} \right)$ (6)

$\frac{\partial^{2}c\left( x_{i}, t \right)}{{\partial x}^{2}}=\delta_{x}^{2}c\left( x_{i}, t \right)-\frac{h^{2}}{12}\frac{\partial^{4}c\left( x_{i}, t \right)}{{\partial x}^{4}}+O\left( h^{4} \right)$ (7)

By use of the control equation, Eq. 1, we obtained:

$\frac{\partial^{3}c\left( x_{i}, t \right)}{{\partial x}^{3}}=\frac{v}{Da}\frac{\partial^{2}c\left( x_{i}, t \right)}{{\partial x}^{2}}-\frac{1}{Da}\frac{\partial f\left( x_{i}, t \right)}{\partial x}$ (8)

$\frac{\partial^{4}c\left( x_{i}, t \right)}{{\partial x}^{4}}=\frac{v^{2}}{{Da}^{2}}\frac{\partial^{2}c\left( x_{i}, t \right)}{{\partial x}^{2}}-\frac{v}{{Da}^{2}}\frac{\partial f\left( x_{i}, t \right)}{\partial x}-\frac{1}{Da}\frac{\partial^{2}f\left( x_{i}, t \right)}{{\partial x}^{2}}$ (9)

By substituting the derivatives in the right hand of Eqs. 8-9 with the central differential schemes, we obtained:

$\frac{\partial^{3}c\left( x_{i}, t \right)}{{\partial x}^{3}}=\frac{v}{Da}\delta_{x}^{2}c\left( x_{i}, t \right)-\frac{1}{Da}\delta_{x}^{1}f\left( x_{i}, t \right)+O(h^{2})$ (10)

$\frac{\partial^{4}c\left( x_{i}, t \right)}{{\partial x}^{4}}=\frac{v^{2}}{{Da}^{2}}\delta_{x}^{2}c\left( x_{i}, t \right)-\frac{v}{{Da}^{2}}\delta_{x}^{1}f\left( x_{i}, t \right)-\frac{1}{Da}\delta_{x}^{2}f\left( x_{i}, t \right)+O(h^{2})$ (11)

By incorporating Eqs. 10-11 into Eqs. 6-7, the compact finite difference schemes of the first and second derivatives can be obtained:

$\frac{\partial c\left( x_{i}, t \right)}{\partial x}=\delta_{x}^{1}c\left( x_{i}, t \right)-\frac{h^{2}v}{6Da}\delta_{x}^{2}c\left( x_{i}, t \right)+\frac{h^{2}}{6Da}\delta_{x}^{1}f\left( x_{i}, t \right)+O\left( h^{4} \right)$ (12)

$\frac{\partial^{2}c\left( x_{i}, t \right)}{{\partial x}^{2}}=\delta_{x}^{2}c\left( x_{i}, t \right)-\frac{h^{2}v^{2}}{12{Da}^{2}}\delta_{x}^{2}c\left( x_{i}, t \right)+\frac{h^{2}v}{12{Da}^{2}}\delta_{x}^{1}f\left( x_{i}, t \right)+\frac{h^{2}}{12Da}\delta_{x}^{2}f\left( x_{i}, t \right)+O\left( h^{4} \right)$ (13)

By substituting Eqs. 12-13 into Eq. 1 and neglecting the error term, a three-point 4th order difference scheme for Eq. 1 can be obtained:

$\left[ -\left( Da+\frac{h^{2}v^{2}}{12Da} \right)\delta_{x}^{2}+v\delta_{x}^{1} \right]c\left( x_{i}, t \right)=\left( \frac{h^{2}}{12}\delta_{x}^{2}-\frac{h^{2}v}{12Da}\delta_{x}^{1}+1 \right)f\left( x_{i}, t \right)$ (14)
